# Spatiotemporal gait parameters and plantar pressure distribution during barefoot walking in people with gout and asymptomatic hyperuricemia: comparison with healthy individuals with normal serum urate concentrations

**DOI:** 10.1186/s13047-016-0147-4

**Published:** 2016-04-30

**Authors:** Sarah Stewart, Nicola Dalbeth, Alain C. Vandal, Keith Rome

**Affiliations:** Department of Podiatry, Health & Rehabilitation Research Institute, Auckland University of Technology, Private Bag 92006, Auckland, 1142 New Zealand; Faculty of Medical and Health Sciences, The University of Auckland, Private Bag 92019, Auckland, 1142 New Zealand; Department of Rheumatology, Auckland District Health Board, P.O. Box 92189, Auckland, New Zealand; Department of Biostatistics & Epidemiology, Faculty of Health and Environmental Sciences, Auckland University of Technology, Private Bag 92006, Auckland, 1142 New Zealand; Health Intelligence & Informatics, Ko Awatea, Counties Manukau Health, Private Bag 93311, Auckland, 1640 New Zealand

**Keywords:** Gout, Asymptomatic hyperuricemia, Gait, Plantar pressure

## Abstract

**Background:**

To identify spatiotemporal gait parameters and plantar pressure distribution during barefoot walking in people with gout and people with asymptomatic hyperuricemia by comparing them to healthy individuals with normal serum urate concentrations.

**Methods:**

Eighty-seven participants were included: 24 with gout, 29 with asymptomatic hyperuricemia and 34 age- and sex-matched normouricemic control participants. Spatiotemporal parameters of gait were assessed during level barefoot walking using a GAITRite® walkway. Peak plantar pressure and pressure time integrals were recorded using a TekScan MatScan®. Results were adjusted for age and body mass index.

**Results:**

Compared to normouricemic control participants, participants with gout demonstrated increased step time (*P* = 0.022) and stance time (*P* = 0.022), and reduced velocity (*P* = 0.050). Participants with gout also walked with decreased peak pressure at the heel (*P =* 0.012) and hallux (*P* = 0.036) and increased peak pressure (*P* < 0.001) and pressure time integrals (*P* = 0.005) at the midfoot. Compared to normouricemic control participants, participants with asymptomatic hyperuricemia demonstrated increased support base (*P* = 0.002), double support time (*P* < 0.001) and cadence (*P* = 0.028) and reduced swing time (*P* = 0.019) and single support time (*P* = 0.020) as well as increased pressure at the midfoot (*P* = 0.013), first metatarsal (*P* = 0.015) and second metatarsal (*P* = 0.007).

**Conclusion:**

During barefoot walking, people with gout walk slower with plantar pressure patterns suggestive of apropulsive and antalgic gait strategies. Individuals with asymptomatic hyperuricemia also demonstrate altered barefoot gait patterns when compared to normouricemic control participants. Clinicians may consider dynamic gait outcomes when assessing and managing foot and lower limb related pain and disability in individuals with gout and asymptomatic hyperuricemia.

## Background

People with gout experience flares of severe inflammatory arthritis as a response to the presence of urate crystals deposited in joints and soft tissues [1, 2]. Gout most commonly affects peripheral structures of the foot and ankle with a propensity for the first metatarsophalangeal joint and Achilles tendon [3, 4]. Although elevated serum urate (hyperuricemia) is required for the development of gout, not all individuals with hyperuricemia develop symptomatic acute gouty arthritis [5]. However, advanced imaging studies have identified urate deposition and joint damage in foot and ankle structures in individuals with asymptomatic hyperuricemia [6–10].

People with gout report chronic and persistent foot and lower limb impairments, even in the absence of acute arthritis [11–16]. Furthermore, foot-related disability has been associated with altered gait parameters in people with gout [16] and may be mirrored in their plantar pressure patterns which have been proposed to reflect pain-avoidance strategies [13]. Individuals with asymptomatic hyperuricemia, who lack any current or previous symptoms of acute gouty arthritis or clinical evidence of urate deposition, also report disabling foot pain and experience lower limb impairments and activity limitations compared to healthy individuals with normal urate levels [14]. However, it is unknown whether their gait and plantar pressure patterns also differ from healthy normouricemic individuals.

Previous plantar pressure research in gout has been undertaken with patients wearing their own footwear [13]. Many people with tophaceous gout of the foot report difficulty in wearing and finding footwear that is appropriate to their level of pain, disability and deformity [17–22]. Footwear worn by people with gout has been shown to be poorly fitting with minimal cushioning and motion control properties [23]. Furthermore, over half of people with gout wear shoes with flexion points proximal to the level of the metatarsal heads which can limit gait efficiency by inhibiting normal first metatarsophalangeal joint function during propulsion [24] and hence may exacerbate the problems of efficient toe-off observed in people with gout during shod walking [13]. Considering the important influence of footwear on foot function, assessment of plantar pressure without the confounding effect of footwear is warranted.

This study aimed to identify spatiotemporal gait parameters and plantar pressure distribution during level barefoot walking in people with gout and people with asymptomatic hyperuricemia by comparing them to normouricemic control participants.

## Methods

### Participants

This investigation was a cross-sectional observational study. Ethical approval for the study was obtained from the Auckland University of Technology Ethical Committee (13/100) and Locality Assessment was obtained from the Auckland District Health Board (ADHB) Research Office (A + 5891). All participants provided written informed consent prior to data collection.

Participants with gout were recruited from the ADHB rheumatology clinic and met the 1977 preliminary American Rheumatism Association (ARA) classification criteria for gout [25]. Participants without gout were recruited from the local community and were to have no history of acute arthritis nor meet the ARA criteria. Participants without gout underwent serum urate testing on the day of the study using a Reflotron® Plus capillary test and were stratified into either the asymptomatic hyperuricemia group (serum urate ≥0.41 mmol/L) or the normouricemic control group (serum urate <0.41 mmol/L, 6.8 mg/dL). The three diagnostic groups were age-and sex-matched. Participants were excluded if they were under 20 years of age; had a history of other inflammatory arthritis; were experiencing an acute flare at the time of the clinical visit; had foot and/or ankle surgery in the previous 3 months; had lower limb amputation; or were unable to walk 5 m unaided.

All data were collected during a single session at the Auckland University of Technology Podiatry Clinic (Auckland, New Zealand) by a single researcher (SS). Demographic data were obtained from all participants including age, gender, ethnicity, body mass index (BMI), current medications and medical history. Peripheral sensation was assessed using a 10 g Semmes-Weinstein monofilament at the plantar hallux, first metatarsal head and fifth metatarsal head. A loss of protective sensation for each foot was defined if absent in at least two of the three sites. Additionally, gout disease characteristics were documented for participants with gout including disease duration, flare history, presence of subcutaneous tophi and tophus count.

### Gait parameters

Spatial and temporal parameters of gait during level barefoot walking were collected using the GAITRite system (CIR Systems, Inc., New Jersey, US). The GAITRite is a 700 cm × 90 cm electronic walkway with an active sensor area of 610 cm long and 60 cm wide. The active area contains 23,040 embedded pressure-activated sensors with a spatial resolution of 1.27 cm and a sampling rate of 120 Hz. All data was processed and stored by an IBM compatible computer using GAITRite® gold, Version 3.2b software. Participants were instructed to walk at their own comfortable walking speed [26] from a point 100 cm before the walkway and finishing 100 cm past its end to ensure that when they reached the walkway they were walking at a normal speed and momentum. Three trials of barefoot walking were recorded for each participant with adequate rest time between trials. Prior to calculation of the gait parameters, the data was reviewed on the monitor screen to ensure that right and left footfalls had been correctly identified, and any footfall not completely on the walkway at either end was removed. For each trial the following temporal and spatial parameters for right and left feet were calculated: velocity (m/s), cadence (steps/min), step length (cm), stride length (cm), support base (cm), step time (s), swing time (s), stance time (s), and single and double support time (s).

### Plantar pressure

**Fig. 1:**
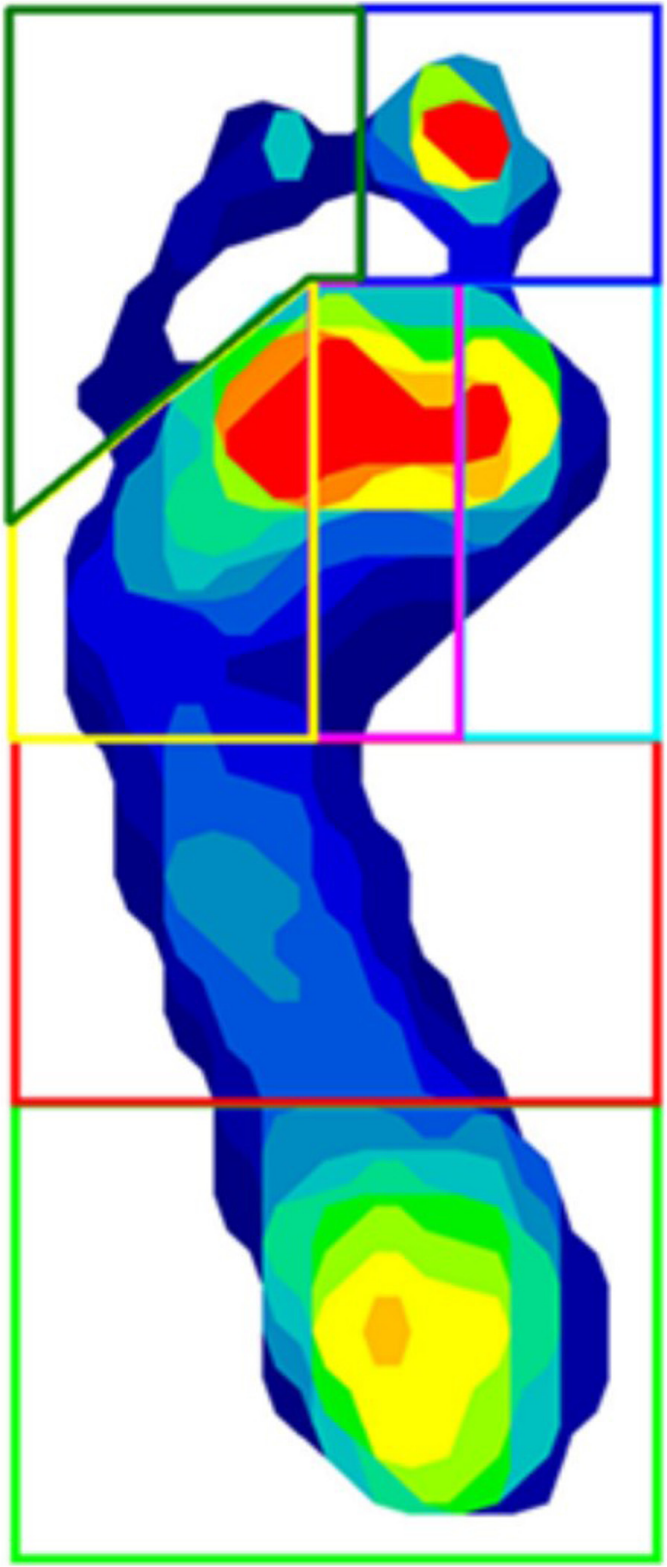
Masking of seven plantar regions

Dynamic plantar pressure measurements were captured during level barefoot walking using the TekScan MatScan® system (Boston, MA, USA). The system consists of a 5 mm thick platform (432 × 368 mm), comprising of 2288 resistive sensors (1.4 sensors/cm^2^) which sample data at a frequency of 40 Hz. Data was collected using the two-step gait initiation protocol [27] which required the participant to step on the platform on their second step. Prior to data acquisition participants were instructed to familiarise themselves with the protocol and line themselves up with the platform to ensure their second step landed in the sensing area. Participants were instructed to walk at their own natural comfortable walking speed and to continue walking past the platform for at least two more steps which ensured that a constant velocity and momentum had been reached and pressure data reflected their normal gait. Three trials were recorded for each foot. The Research Foot® Version 6.61 was used to mask the foot into seven regions representing the heel, midfoot, first metatarsal, second metatarsal, metatarsal three to five, the hallux, and the lesser toes (Fig. 1). This masking method has demonstrated excellent reliability for the calculation of pressure parameters (ICCs 0.96 to 0.99) [28]. Peak plantar pressure (kPa) and pressure time integrals (kPa⋅s) were computed from the software for each masked region.

### Statistical analysis

All continuous outcomes were reviewed for normality using the residuals from a linear model which included relevant demographic covariates and the diagnostic group as the independent variable. Mixed linear regression models were used which accounted for repeated measures taken from right and left feet of each participant in which participant-specific and participant-nested random effects for foot-side were added to the models. This analysis produces results identical to an analysis of measures averaged for each foot-side (if there are no missing values) that allows for a between-foot-side correlation as well as any reweighting required due to missing values. For the spatiotemporal gait parameters the models also accounted for the time-based repeated-measures using a scaled identity repeated covariance structure which assumed the repeated-measures were independent and shared a common variance. For peak plantar pressure and pressure time integrals, which were measured at seven sites on the plantar foot (forming a natural vector of related variables), in addition to the side- and time-based repeated measures, a heterogeneous compound symmetry covariance structure was employed which allowed for separate variances for each site, as well as different covariances (but equal correlations) between each group of sites.

Adjustments for age group, ethnicity and BMI, which were entered into each model simultaneously, were considered only if their level of observed significance achieved at least 10 % on an F-test. These covariates were not expected to behave as confounders due to the frequency matching, but had the potential to decrease residual variance as possible independent variables. Potential covariates were also explored by reviewing box plots of random effects by covariate group.

Two comparisons were considered: gout vs. normouricemic control and asymptomatic hyperuricemic vs. normouricemic control, which were always tested separately. All hypothesis tests (excluding covariate testing) were carried out at a 5 % level of significance against two-sided alternatives. No adjustment for multiplicity was used, but all test-statistics (least-squares (L-S) means), their null distributions and their observed significance levels were reported. Data were analysed using IBM SPSS Statistics version 20 and proc-mixed in SAS version 9.3.

### Sample size

The study was powered to test hypotheses regarding pre-planned analyses comparing normouricemic control parameters with asymptomatic hyperuricemic and gout parameters respectively. With respect to this analysis, the target figures of 21 participants in the gout group, 29 in the asymptomatic hyperuricemia group and 34 in the normouricemic control group are sufficient to detect unadjusted effect sizes on continuous outcomes of 0.7 and 0.8 (moderate to large) between control and each of the asymptomatic hyperuricemia and the gout group, respectively. The achieved sample size improved on these figures.

## Results

### Participant characteristics

A total of 87 participants were included: 24 with gout, 29 with asymptomatic hyperuricemia and 34 normouricemic controls. Demographic and clinical characteristics for the three groups are shown in Table 1. All participants were male with a mean (SD) age of 58 (15) years and predominantly of European ethnicity (*n* = 68, 81 %). The normouricemic control group had a significantly lower mean BMI compared to the gout (*P* < 0.001) and asymptomatic hyperuricemic (*P* < 0.001) groups. The normouricemic control group had a significantly lower prevalence of hypertension compared to the gout (*P* = 0.001) and asymptomatic hyperuricemic (*P* = 0.023) groups and a significantly lower prevalence of cardiovascular disease compared to the gout group (*P =* 0.019). Disease characteristics for the gout group are shown in Table 2. Participants with gout were found to have a mean (SD) disease duration of 17 (11) years, with 71 % (*n* = 17) having tophaceous gout and 96 % (*n* = 23) on urate lowering therapy.

**Table 1:** Demographic and medical characteristics. Data are presented as mean (SD) unless otherwise specified

Variable	Normouricemic control	Gout	Asymptomatic Hyperuricemia
N	34	24	29
Gender, male, *n* (%)	34 (100 %)	24 (100 %)	29 (100 %)
Age, years	58 (14)	58 (13)	58 (19)
Ethnicity, *n* (%)	European 30 (88 %) Māori 1 (3 %) Pacific 0 (0 %) Asian 3 (9 %)	European 14 (58 %) Māori 1 (4 %) Pacific 5 (21 %) Asian 4 (17 %)	European 24 (83 %) Māori 0 (0 %) Pacific 3 (10 %) Asian 2 (7 %)
BMI, kg/m^2^	25.0 (2.9)	30.2 (4.0)†	29.3 (5.9)†
Diuretic use, *n* (%)	4 (12 %)	3 (12 %)	7 (24 %)
Hypertension, *n* (%)	9 (26 %)	17 (70 %)†	16 (55 %)†
Cardiovascular disease, *n* (%)	1 (3 %)	7 (29 %)†	5 (17 %)
Diabetes, *n* (%)	2 (6 %)	4 (17 %)	1 (3 %)
Loss of protective sensation, *n* (%)	Right 1 (3 %) Left 1 (3 %)	Right 4 (17 %) Left 6 (25 %)	Right 4 (14 %) Left 4 (14 %)
Serum urate	0.32 (0.06) mmol/L 5.3 (1.0) mg/dL	0.35 (0.10) mmol/L 5.8 (1.7) mg/dL	0.46 (0.05) mmol/L† 7.6 (0.8) mg/dL

†Significantly different from normouricemic control group (*p* < 0.05)

**Table 2:** Gout disease characteristics. Data are presented as mean (SD) unless otherwise specified

Variable	Gout
Disease duration, years	17 (11)
Age of onset, years	41 (18)
Number of flares in preceding 3 months	1.3 (1.4)
Presence of subcutaneous tophi, *n* (%)	17 (71 %)
Foot tophus count	1.9 (3.5)
Total tophus count	6.1 (8.7)
Colchicine use, *n* (%)	13 (54 %)
Urate lowering therapy, *n* (%)	23 (96 %)

The distribution of residuals from the linear models for all outcome measures demonstrated sufficient normality to carry out parametric testing. The effect of age group and BMI as covariates were included in the final models for all gait and plantar pressure parameters. Table 3 displays the mean estimates and inferential statistics for the spatial and temporal gait parameters. Compared to normouricemic control participants, participants with gout had significantly increased step time (*P* = 0.022), increased stance time (*P* = 0.022) and decreased velocity (*P* = 0.050). Compared to normouricemic control participants, participants with asymptomatic hyperuricemia had significantly increased support base (*P* = 0.002), reduced swing time (*P* = 0.019), decreased single support time (*P* = 0.020), increased double support time (*P* < 0.001) and increased cadence (*P* = 0.028).

**Table 3:** Spatial and temporal gait parameters. Results are presented adjusted for age and BMI

Parameter		Least-squares mean	Diff.	95 % CI		*p*
				Lower	Upper	
Step Length (cm)	Normouricemic control	0.61				
	Gout	0.57	0.03	−0.02	0.08	0.168
	Asymptomatic hyperuricemia	0.61	0.00	−0.05	0.05	0.985
Stride Length (cm)	Normouricemic control	1.21				
	Gout	1.14	0.07	−0.04	0.17	0.200
	Asymptomatic hyperuricemia	1.20	0.02	−0.08	0.11	0.763
Support Base (cm)	Normouricemic control	0.08				
	Gout	0.10	−0.02	−0.03	0.00	0.102
	Asymptomatic hyperuricemia	0.11	−0.03	−0.05	−0.01	0.002
Step Time (s)	Normouricemic control	0.60				
	Gout	0.64	−0.04	−0.07	−0.01	0.022
	Asymptomatic hyperuricemia	0.57	0.03	0.00	0.06	0.081
Swing Time (s)	Normouricemic control	0.46				
	Gout	0.47	−0.02	−0.04	0.00	0.104
	Asymptomatic hyperuricemia	0.43	0.03	0.01	0.05	0.019
Stance Time (s)	Normouricemic control	0.74				
	Gout	0.80	−0.06	−0.10	−0.01	0.022
	Asymptomatic hyperuricemia	0.72	0.03	−0.02	0.07	0.266
Single Support Time (s)	Normouricemic control	0.46				
	Gout	0.48	−0.02	−0.04	0.00	0.092
	Asymptomatic hyperuricemia	0.43	0.03	0.00	0.05	0.020
Double Support Time (s)	Normouricemic control	0.16				
	Gout	0.16	0.00	−0.04	0.04	0.857
	Asymptomatic hyperuricemia	0.26	−0.10	−0.14	−0.06	<0.001
Velocity (m/s)	Normouricemic control	1.03				
	Gout	0.91	0.11	0.00	0.23	0.050
	Asymptomatic hyperuricemia	1.07	−0.05	−0.15	0.06	0.379
Cadence (steps/min)	Normouricemic control	100.9				
	Gout	95.5	5.4	−0.7	11.5	0.080
	Asymptomatic hyperuricemia	107.3	−6.5	−12.2	−0.7	0.028

**Fig. 2:**
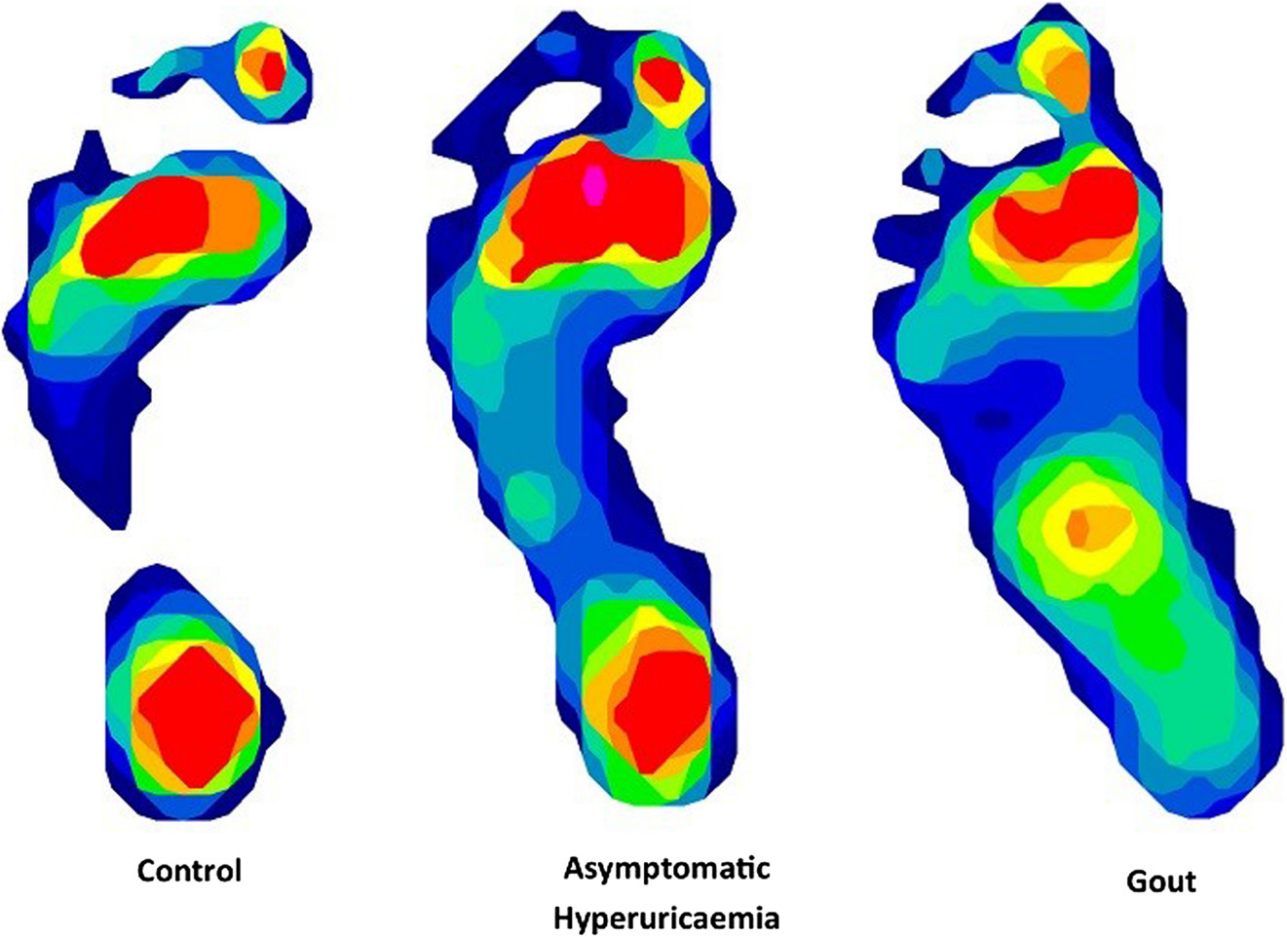
Examples of typical plantar pressure patterns from a control, asymptomatic hyperuricaemia and gout participant

Table 4 displays the mean estimates and inferential statistics for peak plantar pressure. Compared to normouricemic control participants, participants with gout had significantly reduced pressure at the heel (*P* = 0.012) and hallux (*P* = 0.036) and increased pressure at the midfoot (*P* < 0.001). Compared to normouricemic control participants, participants with asymptomatic hyperuricemia had significantly increased pressure at the midfoot (*P* = 0.013), first metatarsal (*P* = 0.015) and second metatarsal (*P* = 0.007). Examples of typical plantar pressure patterns for normouricemic controls and people with asymptomatic hyperuricemia and gout are presented in Fig. 2. Table 5 displays the mean estimates and inferential statistics for the pressure time integrals. Compared to normouricemic control participants, participants with gout had significantly increased pressure time integrals at the midfoot (*P* = 0.006), but no other differences were observed. No differences were observed for pressure time integrals between the asymptomatic hyperuricemic participants and the normouricemic control participants (*P* > 0.05).

**Table 4:** Peak plantar pressure (kPa). Results are presented adjusted for age and BMI

Parameter		Least-squares mean	Diff.	95 % CI		*p*
				Lower	Upper	
Heel	Normouricemic control	294.3				
	Gout	268.2	−26.1	−46.6	−5.6	0.012
	Asymptomatic hyperuricemia	301.9	7.6	−12.4	27.5	0.456
Midfoot	Normouricemic control	95.4				
	Gout	130.8	35.4	15.5	55.3	<0.001
	Asymptomatic hyperuricemia	120.1	24.7	5.3	44.0	0.013
First Metatarsal	Normouricemic control	211.5				
	Gout	229.6	18.2	−5.2	41.5	0.127
	Asymptomatic hyperuricemia	239.7	28.3	5.6	50.9	0.015
Second Metatarsal	Normouricemic control	292.6				
	Gout	287.1	−5.5	−26.9	15.8	0.611
	Asymptomatic hyperuricemia	321.3	28.7	7.9	49.5	0.007
Third to Fifth Metatarsals	Normouricemic control	252.3				
	Gout	244.1	−8.2	−29.6	13.2	0.454
	Asymptomatic hyperuricemia	255.2	2.9	−17.9	23.7	0.785
Hallux	Normouricemic control	233.3				
	Gout	208.4	−24.9	−48.1	−1.6	0.036
	Asymptomatic hyperuricemia	241.9	8.6	−14.0	31.3	0.454
Lesser Toes	Normouricemic control	105.9				
	Gout	121.8	15.9	−2.8	34.6	0.096
	Asymptomatic hyperuricemia	107.2	1.4	−16.9	19.6	0.882

**Table 5:** Pressure time integral (kPa⋅s). Results are presented adjusted for age and BMI

Parameter		Least-squares mean	Diff.	95 % CI		*p*
				Lower	Upper	
Heel	Normouricemic control	61.50				
	Gout	54.68	−6.82	−13.75	0.12	0.054
	Asymptomatic hyperuricemia	59.83	−1.67	−8.44	5.09	0.628
Midfoot	Normouricemic control	23.48				
	Gout	32.66	9.18	2.60	15.76	0.006
	Asymptomatic hyperuricemia	27.17	3.69	−2.72	10.10	0.260
First Metatarsal	Normouricemic control	56.24				
	Gout	54.24	−2.00	−9.50	5.50	0.601
	Asymptomatic hyperuricemia	60.25	4.01	−3.28	11.29	0.281
Second Metatarsal	Normouricemic control	77.61				
	Gout	70.66	−6.95	−14.49	0.59	0.071
	Asymptomatic hyperuricemia	82.15	4.54	−2.80	11.89	0.225
Third to Fifth Metatarsals	Normouricemic control	66.61				
	Gout	61.00	−5.61	−12.90	1.68	0.132
	Asymptomatic hyperuricemia	64.42	−2.18	−9.28	4.92	0.547
Hallux	Normouricemic control	40.66				
	Gout	34.75	−5.91	−13.11	1.29	0.108
	Asymptomatic hyperuricemia	41.74	1.08	−5.94	8.09	0.764
Lesser Toes	Normouricemic control	21.92				
	Gout	23.19	1.28	−4.94	7.49	0.687
	Asymptomatic hyperuricemia	20.48	−1.44	−7.50	4.62	0.642

## Discussion

Our study shows that people with gout and people with asymptomatic hyperuricemia both demonstrate variations in gait parameters and plantar pressure distribution during level barefoot walking when compared to normouricemic control participants.

During barefoot walking, people with gout walked slower with increased time spent in step and stance phases compared to the normouricemic control participants. These findings are consistent with previous research assessing people with gout during both shod and barefoot walking [13, 16, 29]. Reduced gait speed is considered an important characteristic of impaired physical performance in daily activities in adults [30, 31] and has been associated with self-reported foot-related functional limitation in people with gout [16]. Functional gait limitations exhibited by people with gout may result from several factors including reduced lower limb muscle strength [15], loss of normal joint function [14] and acquired gait strategies developed in an attempt to reduce or prevent pain [13, 16].

The increased midfoot and reduced hallux plantar pressures observed in people with gout are also consistent with previous research in which participants were assessed during shod walking [13]. Previous studies have proposed that reduced peak pressure beneath the hallux in people with gout may reflect an attempt to offload pressure at the first metatarsophalangeal joint due to pain [13]. This is further emphasised in qualitative research in which people with gout report attempting to walk more cautiously with an adjusted foot position to relieve the big toe during acute flares [32]. Additionally, inefficient first metatarsophalangeal joint function, previously observed in people with gout [14], may further contribute to the apropulsive gait strategies observed in the current study. In contrast to previous research assessing people with gout during shod walking [13], the current study also observed reduced heel pressures in participants with gout. This is consistent with the slower walking speed and may reflect an attempt to reduce impact at weight acceptance in the absence of protective footwear.

When compared to normouricemic healthy control participants, people with asymptomatic hyperuricemia also exhibited altered gait parameters. They demonstrated an increased base of support, spent more time in double support, less time in single support and swing phases and walked with increased cadence. An increased base of support and double support duration are generally interpreted as adaptions made to produce a more stable and safer gait in older adults who experience mobility limitations [33–35]. The increased cadence also observed in people with asymptomatic hyperuricemia may reflect an attempt to maintain gait velocity while retaining balance and stability. The findings from this study may provide laboratory-based biomechanical support of patient-reported outcomes in which people with asymptomatic hyperuricemia have reported reduced lower limb function and increased activity limitation compared to normouricemic control participants [14].

Participants with asymptomatic hyperuricemia also differed significantly from normouricemic control participants in terms of plantar pressure distribution in which increased pressures in the midfoot and medial metatarsals were observed. Increased midfoot pressures are characteristic of flatter foot postures [36, 37] which have been observed in people with asymptomatic hyperuricemia [14]. This increase in fore- and mid-foot plantar pressure is consistent with that observed in obese individuals [38–40]. However, it should be noted that the analyses in the current study were controlled for BMI. Furthermore, obesity tends to also present with higher toe and heel pressures [38, 40], which were not observed in the current study, suggesting that other factors are driving functional changes in people with asymptomatic hyperuricemia.

The findings from this study should be considered in light of several limitations. Firstly, the participants with gout were recruited from secondary care clinics and may not be representative of those with less severe gout seen in primary care. We did not match groups for BMI, and BMI was higher in the participants with gout and asymptomatic hyperuricemia, compared with the normouricemic control group. Importantly, BMI was included in the analysis models. We cannot exclude the possibility that BMI associated with hyperuricemia had additional unmeasured impact on foot function. Also, we did not exclude participants with diabetes or a loss of peripheral sensation, which may have influenced plantar pressure values in people with gout, reflecting the frequent comorbid conditions observed in clinical practice.

This study highlights the need for future research to provide insight into the dynamic function of the foot, which may assist in the development of interventions for pressure-related foot complaints in people with gout. The relationship between lower limb function during gait and specific locations of joint involvement in gout may also contribute to knowledge in this field of research. An understanding of the impact of comorbid conditions in people with asymptomatic hyperuricemia on functional limitation may also be of interest. The efficacy of non-pharmacological interventions, in combination with pharmacological treatment, aimed at improving lower limb function and patient-reported pain and disability in individuals with gout and asymptomatic hyperuricemia also warrants further investigation.

## Conclusions

In summary, the findings from this study provide novel information regarding plantar pressure distribution during barefoot walking in individuals with gout. The findings are consistent with previous biomechanical research in gout in which patients walk slower with increased midfoot and decreased hallux peak pressures which are suggestive of apropulsive and antalgic gait strategies. This is the first study to assess gait and plantar pressure characteristics in individuals with asymptomatic hyperuricemia. The results have shown that even in the absence of symptomatic gout, people with hyperuricemia exhibit altered gait strategies and plantar loading which may reflect their previously reported high levels of lower limb impairment and disability.

## Ethics, consent and permissions

Ethical approval was obtained for this study and all participants provided written informed consent.

## Abbreviation

BMI: body mass index
